# 3D‐Printed Patient‐Specific Guides Versus Conventional Techniques for Scaphoid Reconstruction: A Comparative Study of 89 Cases

**DOI:** 10.1002/jor.70220

**Published:** 2026-05-03

**Authors:** Sylvano Mania, Mauro Maniglio, Michael A. Wirth, Ladislav Nagy, Lisa Reissner, Andreas Schweizer

**Affiliations:** ^1^ Orthopaedic Department, Balgrist University Hospital University of Zurich Zurich Switzerland

**Keywords:** computer assisted 3‐dimensional planning, osteotomy, patient‐specific instrumentation, scaphoid

## Abstract

Scaphoid non‐union or malunion often produces a three‐dimensional (3D) deformity that alters wrist biomechanics. Patient‐specific instrumentation (PSI) has been introduced to improve reconstruction accuracy, but quantitative comparisons with conventional free‐hand techniques remain limited. We retrospectively analyzed 115 scaphoid reconstructions performed between 2010 and 2019; 89 cases met inclusion criteria (45 PSI, 44 conventional). Distal‐fragment rotation (flexion/extension, ulnar/radial inclination, pronation/supination) and translation were quantified using a Cartesian coordinate system centered on the proximal pole. A composite 3D deformity angle was calculated to assess global malalignment. PSI achieved greater absolute correction across several parameters. Sagittal flexion improved by 8.9° with PSI versus 5.8° with conventional reconstruction (*p* = 0.007 and *p* = 0.043, respectively). Ulnar inclination improved by 3° with PSI (*p* = 0.017) but not significantly with the conventional technique. The composite 3D deformity angle decreased by 8.4° with PSI and 6.9° conventionally. Despite a longer preoperative non‐union duration in the PSI group, consolidation rates (91% vs 93%) and mean times to union were comparable between techniques. Vascularized grafts showed shorter, but non‐significant, consolidation times. Both techniques effectively corrected scaphoid deformity. PSI enabled larger morphological corrections in cases with more severe baseline deformity. These findings suggest that PSI may facilitate correction in complex or long‐standing non‐unions; however, the clinical relevance of the observed 3D differences and operative efficiency remain to be determined.

## 1 Background

Post‐traumatic deformity of the scaphoid is typically characterized by flexion of the distal pole, sometimes with a so‐called humpback configuration, resulting in a collapse of the proximal carpal row. This deformity can be quantified using lateral radiographs, computer tomography (CT) or even echographic measurements [1]. Assessment of scaphoid deformity is generally performed with: the lateral intrascaphoid angle (LISA) obtained by drawing a line through the centres of the proximal and distal poles [2, 3]; the height‐to‐length ratio (H:L) calculated by dividing overall scaphoid length along the palmar cortex by scaphoid height along a perpendicular line to the baseline [2, 4]; the dorsal cortical angle (DCA) with a tangential line connecting the dorsal cortices of the proximal and distal poles [5, 6]; or the anteroposterior intrascaphoid angle (AP‐ISA) [2], which uses the same technique as the LISA. In parallel, CT has enabled a more detailed, three‐dimensional assessment of the deformity, confirming not only the sagittal flexion component but also rotational and coronal deviations of the distal fragment [7].

Whether this post‐traumatic deformity has healed or not, producing respectively a malunion or a non‐union, an alteration of the carpal kinematics can be observed in both cases [8, 9, 10]. While a non‐union leads to a rapid joint degeneration, well known as scaphoid non‐union advanced collapse (SNAC‐Wrist), the clinical impact of scaphoid malunion remains debated [3, 4, 11]. Numerous studies point to an increased risk of post‐traumatic osteoarthritis: a humpback angle > 45° has been linked to degenerative changes in up to 54% of cases despite bony union [2], while a cohort of 229 patients still showed radiocarpal osteoarthritis in 5.2% of malunions [12]. These concerns have driven the development of corrective techniques aimed at restoring dorsal alignment [13, 14].

PSI with 3D‐printed cutting guides have been proposed to improve 3D realignment [15, 16], but their benefit in the anatomical correction over conventional reconstruction remains unclear.

The aim of our study is therefore to quantify three‑dimensional scaphoid deformity and compare the corrective accuracy of 3D‑printed PSI with that of conventional free‑hand reconstruction without PSI.

## 2 Materials & Methods

We conducted a retrospective cohort study of consecutive patients who underwent scaphoid reconstruction at our institution for non‐union or malunion, i.e., healed in a nonanatomic position. From 2010 to 2019, 115 scaphoids were reconstructed by two senior surgeons (LN, AS), level of expertise 5 according to Tang & Giddins [17]. Patients were allocated to one of two cohorts according to the technique used: a historical conventional free‐hand scaphoid reconstruction treated prior the implementation of 3D planning in our clinic, and a PSI group treated after the implementation of PSI technique for scaphoid reconstruction. As our aim was to assess the radiologic accuracy and consolidation, the follow‐up was chosen accordingly. The minimum follow‐up was until confirmation of radiologic consolidation in a CT. In patients without consolidation, the radiological follow‐up was 12 months (SD 5, range 6–17). Table 1 presents patient demographics.

**Table 1:** Patient demographic.

	Technique	
	PSI (*n* = 45)	Conventional (*n* = 44)
Age mean y.o. (SD, min‐max)	29,1 (12, 15–68)	27,2 (10, 13–49)
Weight, mean kg (SD, min‐max)	75 (14, 47–105)	82 (14, 54–116)
Height, mean cm (SD, min‐max)	178 (9, 160–198)	180 (8, 161–198)
Body mass index, mean (SD, min‐max)	23.6 (3.5, 18–34.3)	25.2 (3.1, 19.5–31.6)
Right/left	26/19	22/22
Male/female	41/4	40/4
Time to surgery (months)	47,0	29,4

*Note:* *Missing values for body height/weight for 7 patients (4 with PSI and 3 conventional).

### 2.1 Surgical Techniques

Conventional technique used an iliac cortico‐spongious graft according to the Matti‐Russe technique [14] or with cancellous grafts from radius or iliac crest. Debridement of the callus and the fracture site was performed until bleeding cancellous bone, indicating good vascularity was observed. If bone vascularity was deemed compromised, vascularised cortico‐spongious bone grafts were performed either with a palmar carpal artery graft [18] or with a dorsal 1–2 ICSRA graft [19]. Since the introduction of PSI 3D‐printed cutting guides in our clinic in 2011 relying on the contralateral healthy scaphoid as a template described by Schweizer et al. (2016) [15] and Wirth et al. (2025) [16], most reconstructions have been progressively treated using this new technique. However, during the implementation phase of PSI for scaphoid up until 2018, 25 cases were still treated with the conventional method, when the technique was still under evaluation.

The patient‐specific guides were designed based on preoperative CT data using mirrored contralateral anatomy as a reference. The guides were intended to assist intraoperative orientation and positioning by constraining both the direction and location of the planned corrective cut. The primary guide was shaped to conform to the palmar surface of the scaphoid using predefined anatomical landmarks, ensuring stable and reproducible positioning. It incorporated predefined entry points for Kirschner wires targeting both the proximal and distal poles. Once the guidewires were inserted, the scaphoid was sufficiently stabilized to allow osteotomy and/or debridement of the non‐union site. The initial guide was then removed while maintaining the guidewires in situ. A secondary “repositioning guide” was subsequently mounted over the wires, allowing controlled correction by constraining the orientation of the fragments and maintaining alignment during fixation. Correct positioning of the guides and guidewires was verified intraoperatively using direct visualization of anatomical landmarks and fluoroscopy when required.

The guides were manufactured by Medacta SA (Castel San Pietro, Switzerland) using selective laser sintering with biocompatible polyamide PA2200. Fixation was achieved primarily with anterograde or retrograde headless compression screws, oriented along the longitudinal axis. Conventional non‐headless mini‐fragment screws (1.3 or 1.5 mm) or K‐wires were used when compression was considered inappropriate. Depending on the fracture plane and the available space, two implants (conventional screws and/or K‐wires) could be inserted in different orientations, with one implant along the classical longitudinal axis and another placed in a retrograde vertical direction. Details of the surgical techniques used in both cohorts are presented in Table 2. Postoperatively, patients underwent plaster immobilization for 2 months, followed by a CT scan to determine whether they could begin mobilization.

**Table 2:** Surgical technique.

			Technique	
			PSI	Conventional
Number of implants	None		0	2
	1	Herbert screw	43	39
		Kirschner‐Wire	0	1
	2	Herbert screw	0	1
		Kirschner‐Wire	1	0
		Conventional screw	0	1
		Herbert + conventional	1	0
Bone graft	Palmar carpal artery bone		20	7
	1‐2 ICSRA		7	18
	Non vascularized	Iliac crest	11	13
		Radius	4	3
	Kuhlmann + cancellous	Iliac crest	0	0
		Radius	0	1
	No graft		3	2

*Note:* Shows the surgical details of the two cohorts.

Abbreviations: ICSRA = intercompartimental supra‐retinacular artery, PSI = patient‐specific instrumentation.

### 2.2 Mean 3D Scaphoid Model Shape

**Figure 1:**
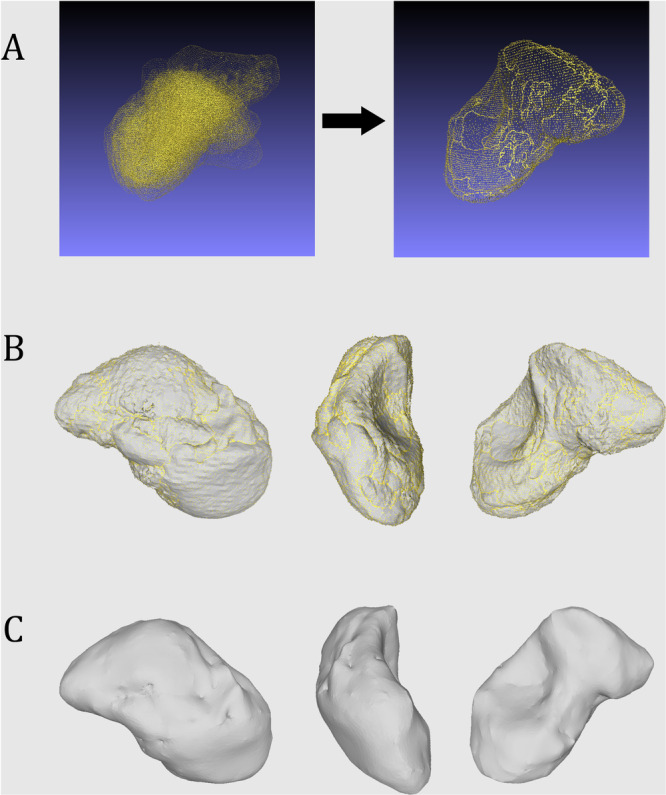
Generation of the mean 3D shape model of a scaphoid. (A) Cloud representation of multiple 3D surface scaphoid model before and after manual alignment and Boolean fusion, based on scaphoid CT of 17 patients. (B) Radial, dorsal and ulnar view of 3D surface scaphoid model after fusion. (C) Final model after edge smoothing.

As bilateral pre‐operative CT was not routinely performed before the introduction of PSI scaphoid reconstruction, the conception of a mean 3D scaphoid model shape was mandatory to perform a pre‐ and postoperative deformity analysis of the patient operated with the conventional technique. Patients were retrospectively and randomly selected from a database of individuals who required patient‐specific cutting guide based on the healthy side, for hand or wrist surgery. CT from 17 patients with healthy wrists were selected: 7 left and 10 right, 15 males and 2 females, mean age 31.6 years old (SD 13.6, min‐max 18.1–68.1). 3D surface models have been generated using Mimics software (Materialise, Leuven, Belgium). The lunate bones have been manually rescaled and used as anatomical reference for size uniformization of the wrist. The 3D surfaces were then exported in Meshlab (version 2023.12, Visual Computing Lab, ISTI‐CNR, Pisa, Italy) to perform manual alignment of the scaphoid 3D model by a single trained observer (SM) to minimize procedural variability. Once an adequate alignment of the scaphoid ridge, the scapho‐capitate articular surface, the proximal and distal pole was reached, a Boolean fusion was performed. Further edge smoothing of the scaphoids was performed in our in‐house planning software (CASPA, Balgrist CARD AG, Zürich, Switzerland) with a 170° smoothing angle and 500 iterations. (Figure 1).

### 2.3 Deformity Analysis

Consolidation was defined as > 65% bone bridging on CT, first assessed at 8 weeks and, if absent, re‐evaluated every 6–8 weeks. Morphological analysis was performed on the last pre‐operative and initial postoperative CT to best reflect immediate surgical accuracy.

**Figure 2:**
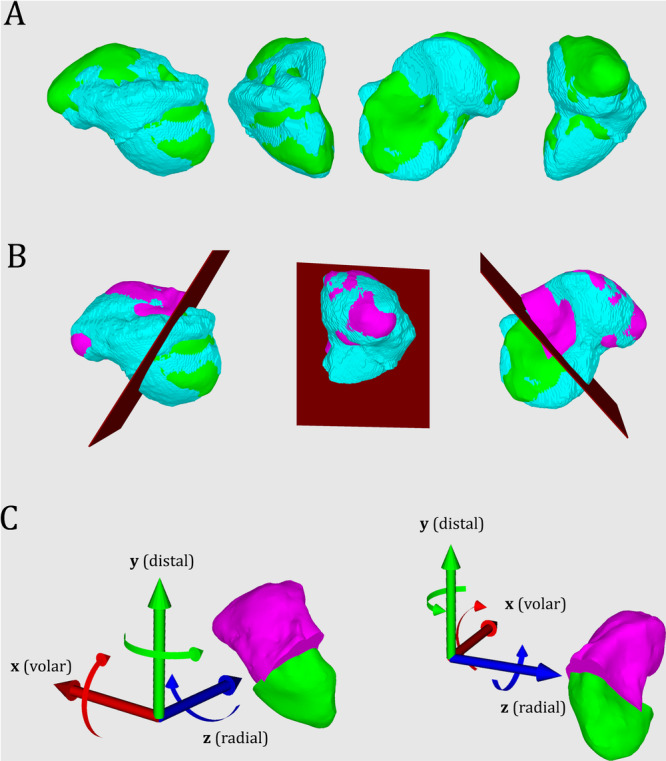
Method of 3D deformity analysis. Figure 2 shows an example of the deformity analysis of a right scaphoid malunion in the waist. (A) Alignment at the proximal pole of the pathological scaphoid (cyan) and the healthy 3D shape model (green) on their proximal pole. (B) Cutting of the healthy scaphoid on its proximal third and alignment of the distal fragment (pink) at the distal pole of the pathological scaphoid (cyan). (C) 3D cartesian coordinates along the dorsal‐to‐volar (x axis), proximal‐to‐distal (y axis) and radial‐to‐ulnar plane (z axis), used as reference for deformity analysis.

The healthy 3D scaphoid shape model has been rescaled to the pre‐ and postoperative surface model of each pathological scaphoids, mirrored when necessary to correspond to the correct side, and aligned at the proximal pole following by minimizing the distance between corresponding surfaces, following the principle of iterative closest point registration, but manually and visually performed by a single observer. A fragmentation either at the proximal or distal third of the healthy scaphoid was performed and its distal pole aligned to the distal pole of the pathological. Once adequate superposition was achieved, the rotation and translation were recorded within a fixed 3D Cartesian coordinate system placed along the proximal‐to‐distal, ulnar‐to‐radial and dorsal‐to‐volar plane. (Figure 2). All the manual alignment process were performed through a single observer (SM) using a standardized workflow. An additional analysis of the overall deformity was performed using a three‐dimensional composite angle (3D angle), incorporating the cumulative deviation across all rotational planes. The 3D deformity was calculated for each case using the formula:

$$3D\;\mathrm{angle}\;=\sqrt{{\left(\text{Rot X}\right)}^{2}{\;+\;(\text{Rot Y})}^{2}+{\left(\text{Rot Z}\right)}^{2}})$$

*where **Rot X** represents ulnar–radial inclination, **Rot Y** represents supination–pronation, and **Rot Z** represents flexion–extension.*

### 2.4 Statistical Analysis

Statistical analysis was performed using IBM SPSS, version 26 (SPSS Inc., Chicago, IL). Normality was assessed using the Shapiro‐Wilk test, as well as by evaluating skewness and kurtosis. Although the Shapiro‐Wilk test indicated significant deviations from normality (*p* < 0.05) for a few variables (ulnar‐to‐radial translation in PSI group, supination‐pronation and flexion‐extension in conventional group), the observed skewness values remained within −0.62 and +0.57, and kurtosis values were all below 1.61, which are considered acceptable thresholds. Given the sample sizes and the robustness of ANOVA to minor deviations from normality, parametric analyses were considered appropriate. Homogeneity of variances was confirmed using Levene's test (*p* > 0.05 for all variables). Consequently, one‐way ANOVA was used for all group comparisons, with statistical significance set at *p* < 0.05. For the 3D angular deformity, however, the Shapiro–Wilk test confirmed clear non‐normal distribution, while Levene's test indicated homogeneous variances. Consequently, the non‐parametric Mann–Whitney test was used for pre‐ and postoperative group comparisons of 3D Angle, with statistical significance set at *p* < 0.05.

An inter‐group analysis of pre‐operative deformity distribution was conducted by categorizing scaphoid rotations along the three anatomical planes (flexion–extension, ulnar–radial inclination, and pronation–supination) into 5° intervals, and by categorizing the translation of the distal pole (proximal‐distal, volar‐dorsal, radial‐ulnar) into 2 mm intervals. Distributional variability between the PSI and conventional groups was evaluated both pre‐ and postoperatively using Chi‐squared tests of independence for each rotational axis.

For the analysis of time to consolidation between the PSI and conventional techniques, patients were stratified according to the type of bone graft used: palmar vascularised graft (Kuhlmann type), dorsal 1–2 ICSRA graft (Zaidemberg type), non‐vascularised graft, and no graft. A Shapiro–Wilk test indicated a non‐normal distribution (*p* < 0.05) for all groups except the “no‐graft” subgroup. However, given the very small sample size in this subgroup (*n* = 4), no formal statistical comparison was performed, and only descriptive data are reported.

## 3 Results

Of 115 reconstructions originally identified (63 PSI, 52 conventional), 26 cases did not meet the technical requirements for 3D evaluation: 17 PSI cases lacked convertible historical planning files, and 8 free‑hand cases had CT of insufficient quality for modelling. In addition, one PSI patient sustained a new fracture 13 months after achieving consolidation at 57 days and required a second reconstruction; only the index procedure was retained for morphological analysis. The final analysis therefore included 45 PSI and 44 conventional reconstructions, yielding two well‑balanced cohorts for quantitative comparison.

The distribution of primary and revision cases was similar between groups. In the PSI cohort, 32/45 (29%) were revision procedures, compared with 13/44 (30%) in the conventional cohort, indicating no relevant difference in case complexity between techniques.

### 3.1 Consolidation

The PSI group had a mean duration of non‐union prior to surgery of 47.8 months (SD 71.2, range 3–400), compared with 29.5 months (SD 55, range 1–321) in the conventional group. Consolidation was achieved in 43 of 45 cases (95.5%) in the PSI group and 42 of 44 (95.5%) in the conventional group. Mean time to consolidation was 117 days (SD 154, range 29–1050) for PSI and 130 days (SD 113, range 49–615) for conventional reconstructions. No statistically significant difference in consolidation time was observed between graft types or between reconstruction techniques (PSI or conventional). We note however that among five cases without bone graft, 1 out of 3 cases showed no consolidation in the PSI group and 2 out of 2 in the conventional group healed, with a mean time to consolidation of respectively 102 days (SD 16, range 91–113) versus 54 days (SD 1, range 53–54). Vascularised bone grafts, whether palmar‐ or dorsal‐based, demonstrated shorter but non‐significant consolidation times compared with non‐vascularised grafts. Palmar‐based grafts consolidated after a mean of 107 days (SD 60, range 49–259), with 112 days for PSI and 91 days for conventional procedures (*p* = 0.583). Reconstructions using a 1–2 ICSRA graft consolidated after a mean of 113 days (SD 73, range 51–334), with 92 days for PSI and 120 days for conventional techniques (*p* = 0.194). Non‐vascularised grafts showed consolidation times, averaging 150 days (SD 205, range 53–1050), with 145 days for PSI and 156 days for conventional reconstruction (*p* = 0.322). (Table 3).

**Table 3:** Consolidation.

			Bone graft														
			Palmar‐based (Kuhlmann)					Dorsal‐based 1‐2 ICSRA (Zaidemberg)					Non vascularized				
			Mean	Min	Max	SD	*n*	Mean	Min	Max	SD	*n*	Mean	Min	Max	SD	*n*
With PSI	Time to surgery (month)		49,11					37,29					17,67				
	Time to consolidation (days)		105	29	259	64		92	51	178	51		142	53	1050	253	
	Consolidation	N					0					1					0
		Y					20					6					15
Conventional	Time to surgery (month)		13,14					35,88					33,07				
	Time to consolidation (days)		91	49	177	49		124	54	343	78		156	54	615	158	
	Consolidation	N					1					1					0
		Y					6					17					16
All techniques	Time to surgery (month)		39,04					36,30					25,37				
	Time to consolidation (days)		102	29	259	60		116	51	343	73		149	53	1050	206	
	Consolidation	N					1					2					0
		Y					26					23					31

			Bone graft														
			Kuhlmann + cancellous					No graft					All graft technique				
			Mean	Min	Max	SD	*n*	Mean	Min	Max	SD	nt	Mean	Min	Max	SD	*n*
With PSI	Time to surgery (month)							203,33					46,98				
	Time to consolidation (days)							102	91	113	16		116	29	1050	154	
	Consolidation	N					0					1					2
		Y					0					2					43
Conventional	Time to surgery (month)		43,00					1,00					29,44				
	Time to consolidation (days)		194	194	194			54	53	54	1		130	49	615	113	
	Consolidation	N					0					0					2
		Y					1					2					42
All techniques	Time to surgery (month)		43,00					122,40					38,42				
	Time to consolidation (days)		194	194	194			78	53	113	29		123	29	1050	135	
	Consolidation	N					0					1					4
		Y					1					4					85

*Note:* Shows time to consolidation and consolidation rate according to surgical techniques with or without.

Abbreviations: ICSRA = intercompartimental supra‐retinacular artery, N = no, PSI = patient‐specific instrumentation and depending on bone graft, Y = yes.

### 3.2 Deformity Correction

**Figure 3:**
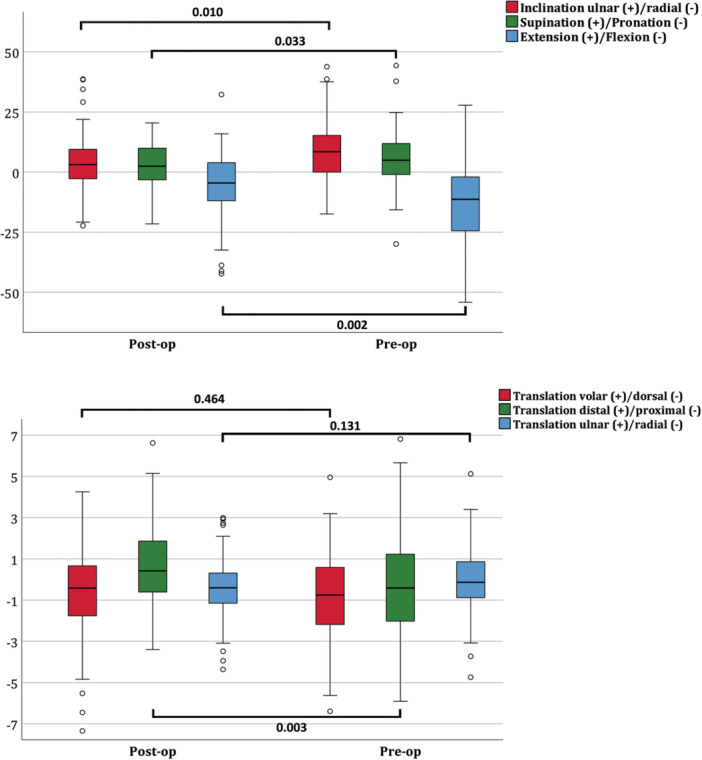
Deformity correction with or without patient‐specific instrumentation. Figure 3 shows the Rotational and translational variation of the distal scaphoid pole. Boxplots show changes in ulnar–radial inclination or volar–dorsal translation (red), pronation–supination or proximal–distal translation (green), and flexion–extension or ulnar–radial translation (blue) before (Pre‐op) and after (Post‐op) reconstruction using either patient‐specific instrumentation (PSI) or conventional free‐hand technique. Whiskers represent the 95% confidence interval, and p‐values indicate statistical significance (*p* < 0.05) for within‐group pre‐ to postoperative comparisons.

PSI reconstruction produced larger absolute corrections across key deformity parameters, reflecting its use in cases with greater preoperative deformity (Figure 3). Sagittal flexion improved by 8.9° (from 20° to 11°, *p* = 0.007), compared with 5.8° in the conventional group (from 5° to 0°, *p* = 0.043). Distal–proximal length improved by 1.0 mm with PSI (–0.39 mm to +0.65 mm, *p* = 0.057) versus 0.9 mm conventionally (–0.40 mm to +0.49 mm, *p* = 0.014). Ulnar inclination improved by 3° with PSI (5° to 2°, *p* = 0.017), while the 4° reduction in the conventional group (10° to 6°) was not statistically significant (*p* = 0.142). PSI also achieved a greater reduction of the composite 3D deformity angle, improving by 8.4° (26.9° to 18.5°, *p* = 0.002) compared with 6.9° in the conventional group (24.8° to 17.9°, *p* = 0.012).

Translations in the dorsal–volar and ulnar–radial directions showed minimal change in both groups. (Figure 3) Although PSI enabled larger corrections, postoperative dispersion remained substantial, particularly in the sagittal plane.

The Chi‐squared analysis of pre‐operative intergroup variability revealed a significant change in the distribution of flexion–extension values between the pre‐ and postoperative states (*χ*² = 24.29, df = 12, *p* = 0.019), indicating inhomogeneity in this parameter because a much larger pre‐operative flexion in the PSI group with 20° (CI 15.23–25.02) vs 5° (CI 0.78–10.11). A preoperative flexion deformity (defined as > 0°) was observed in 41 of 45 cases (91%) in the PSI group, including 38 (84%) with flexion greater than 5°, whereas in the conventional group, 30 of 44 cases (68%) showed flexion, and 21 (48%) had flexion greater than 5°. In contrast, ulnar–radial inclination (*χ*² = 14.87, df = 13, *p* = 0.315) and pronation–supination (*χ*² = 18.1, df = 11, *p* = 0.079) and proximal‐distal translation of the distal pole (*χ*² = 6.01, df = 7, *p* = 0.539) showed no significant distributional differences, suggesting pre‐operative homogeneity between the PSI and conventional groups for these three parameters. The proportion of cases with more than 5° of flexion deformity decreased postoperatively from 84% to 67% in the PSI group, and from 48% to 32% in the conventional group.

Postoperative dispersion differed most clearly in the sagittal plane, with PSI ranging from 42.3° flexion to 8.2° extension, whereas the conventional technique spanned 27.9° flexion to 32.3° extension, showing a greater tendency toward over‐extension without PSI. Proximal–distal translation ranged from 3.3 mm shortening to 6.6 mm lengthening with PSI versus 3.4 mm shortening to 3.6 mm lengthening conventionally. Volar–dorsal translation also varied more widely with PSI (7.3 mm dorsal to 4.3 mm volar) compared with the conventional technique (3.1 mm dorsal to 3.0 mm volar). The cumulative 3D angular deformity ranged from 0.5° to 43.1° with PSI and 3.4° to 42.9° conventionally, reflecting similar maximal global deviations. Coronal and axial rotations showed comparable postoperative ranges between techniques.

## 4 Discussion

We conducted a comparative analysis of the morphological outcomes of scaphoid malunion reconstruction performed with PSI 3D‐cutting guides versus conventional techniques.

Both surgical techniques achieved meaningful correction of the scaphoid deformity. Flexion decreased significantly in each group, ulnar inclination was reduced more effectively with PSI, and conventional reconstruction produced a slightly greater gain in distal‑pole length. Pronation–supination changed little, consistent with the minimal pre‑operative malalignment observed in that plane.

Although the PSI group contained a higher proportion of pronounced flexion deformities preoperatively, both techniques substantially reduced the number of cases exceeding 5° of flexion. In the sagittal plane, the conventional group displayed a wider postoperative spread, driven by cases of over‐extension, while proximal–distal and volar–dorsal translations showed slightly broader ranges with PSI. These differences likely reflect the wider spectrum of preoperative deformities treated with PSI rather than reduced technical precision. Overall, both methods achieved comparable consistency of alignment in the coronal and axial planes, with similar cumulative 3D angular deviations. Taken together, these results suggest that PSI may facilitate correction in cases with pronounced sagittal deformity.

Optimal use of PSI guides may require extensive scaphoid dissection to expose anatomical landmarks, raising concerns about potential effects on vascularity. Nevertheless, our cohort demonstrated a high consolidation rate despite the PSI group including longer standing non‐unions. Furthermore, when a dorsal approach using a 1–2 ICSRA vascularised bone graft was performed, PSI reconstructions achieved consolidation times comparable to those obtained with palmar‐based pedicled grafts. Although not statistically significant, it is noteworthy that non‐vascularised bone grafts demonstrated longer consolidation times overall.

Recent clinical and radiological evidence further indicates that this technique is effective not only in primary but also in revision procedures by providing similar consolidation rate, range of motion and grip strength [16]. Our study further adds that consolidation outcomes were comparable between the PSI and conventional groups, reinforcing the evidence that both techniques achieve similar union rates despite differences in case complexity.

Scaphoid non‐union can alter carpal kinematics by uncoupling the proximal and distal carpal rows [10], because of carpal collapse associated with the development of a DISI deformity. In‐vitro cadaveric studies have also demonstrated that scaphoid malunion leads to kinematic changes, notably a progressive extension of the lunate [9]. Computational models simulating scaphoid malunion with flexion deformity have further shown an ulnarly translated and broader contact area within the radiocarpal joint, with increased volar contact during wrist extension [8]. Although a consistent clinical correlation has not been demonstrated in patient‐reported outcomes [11], the combination of post‐traumatic osteoarthritis risk [2] and the documented kinematic alterations provides, in the authors' opinion, sufficient rationale to support anatomical correction of scaphoid deformity, particularly in younger patients. In line with this biomechanical rationale, our findings indicate that PSI‐assisted reconstruction achieved a greater restoration of scaphoid height along the proximal–distal axis compared with the conventional technique. Although this greater correction may theoretically influence carpal alignment and prevent progressive DISI deformity, the present study did not assess functional outcomes or long‐term degeneration, and no clinical inference can be made.

A key strength of this work is the novel Cartesian, three‑dimensional measurement system anchored to the distal pole. Although rarely described previously, an all‑plane, fully 3D assessment provides more precise quantification than traditional two‑dimensional methods, such as LISA, height‑to‑length ratio, DCA, or AP‑ISA, which have shown moderate reproducibility [2, 6].

In a previous CT‐based work with series of 11 cases, Schweizer et al. (2012) [7] reported a mean flexion deformity of 23° (SD 12), ulnar deviation of 5° (SD 12), pronation deformity of 10° (SD 6), and proximal translation of 3.3 mm (SD 3.5). The present cohort showed lower average flexion (13°) and markedly less proximal translation (0.4 mm) while documenting a comparable ulnar deviation (9°) and an unexpected net supination (6°). Although humpback deformity is the most frequent pattern, it is not always pronounced, and distal pole extension was observed in 11 patients (> 5°). These differences likely reflect sample size, and to our knowledge, this study provides the largest 3D description of scaphoid malunion deformity and the first direct comparison of conventional versus PSI reconstruction.

### 4.1 Limitations

First, the retrospective design prevented randomisation; the PSI group started with much greater flexion, and no propensity matching was performed, so residual selection bias cannot be excluded. No a priori sample size calculation was performed, as this retrospective study was exploratory. Based on the final sample size (45 PSI, 44 conventional), the study had 80% power to detect a between‐group effect size of Cohen's d ≈ 0.60 (α = 0.05), so smaller differences may have remained undetected. Multiple between‐group comparisons were conducted, increasing the risk of type‐I error. Second, the cohort was overwhelmingly male, young with normal body mass index, thus with good bone quality, limiting generalisability. However, this demographic distribution reflects the population most affected by this pathology. Third, the small differences observed between cohorts may partly reflect surgical expertise, as all procedures were performed by experienced high‐volume surgeons, likely contributing to the favourable outcomes in the conventional group. Whether outcome improvements between PSI and conventional can be achieved by lower‐volume surgeons remains to be determined. Operative time and fluoroscopy exposure was not systematically recorded and therefore could not be compared in this study. However, the PSI technique assumed to be easier to perform by the authors, may make this procedure more accessible to less specialized or lower‐volume surgeons. All 3D analysis were performed manually by a single trained observer to minimize procedural variability. Inter‐ and intraobserver reliability were not assessed, which represents a limitation of the present study, particularly for small angular differences. The use of a mean scaphoid model derived from a limited number of healthy individuals represents a limitation. Although size differences were addressed by scaling the model using the lunate as a reference, anatomical variability was not specifically accounted for. Further studies with larger normative datasets may further improve accuracy.

While the present study focuses solely on quantitative morphological correction and bone consolidation, clinical outcomes such as range of motion, grip strength, and patient‐reported function were not evaluated and should be investigated in future prospective studies. A key limitation of this work is that the clinical benefits of PSI cannot be balanced against the additional costs incurred, as no functional or cost–benefit analysis was performed. In our clinical workflow, however, production time has been substantially reduced through close and efficient collaboration with the engineering team, allowing a mean guide fabrication time of approximately 4 to 6 weeks following CT acquisition. The cumulative cost of the CT, 3D printing, and sterilisation of the guides is estimated at approximately 3000 CHF, which appears acceptable considering the gain in precision and reproducibility in complex cases.

In conclusion, both conventional and PSI‐guided reconstructions provided reliable correction of scaphoid deformity and high consolidation rates. PSI achieved greater precision in sagittal and coronal realignment without compromising bone healing, despite being used in more challenging, long‐standing non‐unions. These findings support further investigation of patient‐specific guides as a tool for assisting correction in complex scaphoid deformities, while prospective studies incorporating functional and economic outcomes are required.

## Author Contributions

Original idea: L.R., A.S., and L.N. Data curation: S.M., M.M., M.W. Formal analysis: S.M., M.W. Statistical analysis: S.M., Writing of original draft: S.M., Illustration: S.M., Manuscript review: M.M., L.R., A.S. Supervision: M.M., L.R., A.S. All authors reviewed and edited the manuscript and approved the final version of the manuscript.

## Ethics Statement

Ethical approval for this study was obtained the 22.12.2020 from The Cantonal Ethic Committee Zurich (W859/BASEC Nr. 2020‐02860).

## Conflicts of Interest

The authors declare no conflicts of interest.

## Data Availability

The data that support the findings of this study are available from the corresponding author upon reasonable request.
